# Non-CpG Oligonucleotides Exert Adjuvant Effects by Enhancing Cognate B Cell-T Cell Interactions, Leading to B Cell Activation, Differentiation, and Isotype Switching

**DOI:** 10.1155/2015/340468

**Published:** 2015-08-25

**Authors:** Melinda Herbáth, Krisztián Papp, Anna Erdei, József Prechl

**Affiliations:** ^1^MTA-ELTE Immunology Research Group, 1/C Pázmány Péter Sétány, Budapest 1117, Hungary; ^2^Department of Immunology, Eötvös Loránd University, 1/C Pázmány Péter Sétány, Budapest 1117, Hungary

## Abstract

Natural and synthetic nucleic acids are known to exert immunomodulatory properties. Notably, nucleic acids are known to modulate immune function via several different pathways and various cell types, necessitating a complex interpretation of their effects. In this study we set out to compare the effects of a CpG motif containing oligodeoxynucleotide (ODN) with those of a control and an inhibitory non-CpG ODN during cognate B cell-T cell interactions. We employed an antigen presentation system using splenocytes from TCR transgenic DO11.10 mice and the ovalbumin peptide recognized by the TCR as model antigen. We followed early activation events by measuring CD69 expression, late activation by MHC class II expression, cell division and antibody production of switched, and nonswitched isotypes. We found that both of the tested non-CpG ODN exerted significant immunomodulatory effects on early T cell and on late B cell activation events. Importantly, a synergism between non-CpG effects and T cell help acting on B cells was observed, resulting in enhanced IgG production following cognate T cell-B cell interactions. We propose that non-CpG ODN may perform as better adjuvants when a strong antigen-independent immune activation, elicited by CpG ODNs, is undesirable.

## 1. Introduction

Pathogen associated molecular patterns (PAMPs) activate antigen presenting cells (APCs) via pattern recognition receptors (PRRs) and this process is required for the development of an efficient immune response against the pathogens. Bacterial DNA and synthetic oligonucleotides like CpG oligonucleotides [1] are one of the classes of PAMPS that stimulate cells via PRRs. Besides TLR9 several proteins have been described as the candidate receptors for oligodeoxynucleotides (ODNs), such as CD14 [2], membrane bound scavenger receptors like CXCL16 [3] or SR-A and MARCO [4], DEC-205 [5], human CR2 [6], the KIR3DL2 receptor on human NK cells [7], and alpha 2-macroglobulin [8]. It has also been proposed that the uptake of ODN that have a phosphorothioate (PS) backbone differs from that of natural phosphodiester backboned ODN, and PS ODN bind to many proteins due to nonspecific interactions [9, 10], with no consensus about the exact pathways mediating cellular entry, docking, and signalling induced by these ODNs. Recent studies demonstrated that various TLRs, among them TLR9, are also expressed in different murine and human T cell subsets and have costimulating function. In combination with TCR activation, TLR9 ligands have been shown to induce cytokine production and to promote survival [11, 12]. However, TLR9 cannot be solely responsible for this phenomenon, as T cells of TLR9 or MyD88 deficient mice also respond to CpG and interestingly, even to certain non-CpG ODNs, including inhibitory ODNs [13].

Therefore, it would be very important to understand how T cells and B cells costimulated with non-CpG ODN contribute to the development of an adaptive immune response. Our aim was to investigate how non-CpG ODN modulates antibody production following cognate interaction of T cells with B cells presenting antigen. To this end we examined early and late activation events leading to isotype switching in B cells, a process that enables a more effective host defense against pathogens.

## 2. Materials and Methods

### 2.1. Ethics Statement

All the treatments of animals (mice) in this research followed the guidelines of the Institutional Animal Care and Ethics Committee at Eötvös Loránd University that operated in accordance with permissions 22.1/828/003/2007 issued by the Central Agricultural Office, Hungary, and all animal work was approved by the appropriate committee.

### 2.2. Animals and Cell Culturing

BALB/c mice were purchased from Charles River Laboratories; DO11.10 mice (on the BALB/c background) were derived from The Jackson Laboratory. Both strains were bred and maintained under specific pathogen free conditions in the animal unit of the Eötvös Loránd University. Mice were used at 6–18 wk of age. Spleen or lymph node cell suspensions were cultured in RPMI 1640 medium (GIBCO, Invitrogen, Carlsbad, CA, US) supplemented with 5% heat-inactivated FCS (GIBCO), 2 mM L-glutamine (Sigma-Aldrich, St. Louis, MO, US), 100 U/mL penicillin (Sigma-Aldrich), 100 *µ*g/mL streptomycin (Sigma-Aldrich), 50 *µ*M 2-mercaptoethanol (Sigma-Aldrich), and 1 mM sodium pyruvate (Reanal, Budapest, HU).

### 2.3. Oligodeoxynucleotides and Ovalbumin Derived Peptide

All oligodeoxynucleotides (ODN) had phosphorothioate (PS) linkages between the nucleobases (marked with capital letters), except the one preceding the last two 3′ base (marked with lowercase letters). CpG (ODN 1668; TCCATGACGTTCCTGATGCt), Control (ODN 1720; TCCATGAGCTTCCTGATGCt), and Inhibitor (ODN 2088; TCCTGGCGGGGAAGt) were purchased from Sigma-Aldrich. The ovalbumin derived peptide (OVA) with the following sequence Biotin-KISQAVHAAHAEINEAGR was synthesized by the CASLO Laboratory ApS (Lyngby, Denmark).

### 2.4. *In Vitro* Cell Activation

Freshly isolated spleen or lymph node cells (pooled from the subiliac, popliteal, proper and accessory axillary, superficial parotid, mandibular, and sciatic lymph nodes) were plated onto 96-well plates in 2 × 10^5^ cells/well density. Inhibitor, Control, and CpG ODNs were added in low (0.25 *µ*M) or high (2.5 *µ*M) concentrations with or without a suboptimal activating dose (25 nM) of OVA peptide. Cells were incubated for 1 day at 37°C in 5% CO_2_ humidified atmosphere and CD69 expression was measured or cultured for 2 days with addition of 5-ethynyl-2′-deoxyuridine (EdU) on the first day, and the percent of divided cells was measured on the second day using a “click” reaction assay, according to the manufacturer's protocol (Click-iT EdU flow cytometry assay kit, Invitrogen). MHCII expression or number of antibody secreting cells was determined with flow cytometry or fluorescent ELISPOT, respectively, from 4-day cultures.

### 2.5. Flow Cytometry

Fluorescently labeled mAbs obtained from eBioscience (San Diego, CA, US) were the following: anti-mouse CD45R-PerCP-Cy5.5 (clone: RA3-6B2), rat IgG2a-PerCP-Cy5.5 isotype control (clone: eBR2a); BD Biosciences (Franklin Lakes, New Jersey, US): anti-mouse CD4-PE (clone: (L3T4) (RM4-5)), rat anti-mouse I-A/I-E-PE (clone: M5/114.15.2), rat IgG_2b_, *κ*-PE isotype control; BioLegend (San Diego, CA, US): anti-mouse CD69-A647 (clone: H1.2F3). For cell surface staining, cell suspensions were incubated on ice for 20 min with different combinations of mAb, diluted in FACS buffer (PBS supplemented with 1% heat-inactivated FCS and 0.1% sodium azide). Nonspecific binding was blocked using heat inactivated mouse serum in 4-time dilution. After staining with fluorescently labeled mAb, cells were washed and acquired by a FACSCalibur (BD Biosciences) flow cytometer and results were analyzed using FCSExpress (De Novo Software). In CD45R^+^CD4^−^ lymphocyte-sized cells were considered B cells and CD45R^−^CD4^+^ lymphocyte-sized cells were considered T cells. Dead cells were excluded on the basis of their light scattering properties.

### 2.6. Fluorescent ELISPOT

16-pad nitrocellulose-covered glass slides (UniSart, Sartorius Stedim Biotech, Goettingen, Germany) were put into slide modules (ProPlate, Grace Bio-Labs, Bend, OR, USA) and rinsed with PBS for 5 min before use and coated overnight with 2.5 *µ*g/mL anti-mouse kappa capture antibody (Southern Biotech, Birmingham, AL, USA) in PBS. Slides were then washed with PBS three times and blocked for 1 hour at 37°C with RPMI-1640 medium supplemented with 10% FCS. For the measurement of IgG and IgM antibody secreting cells (ASCs) 2/3 volume for the measurement of IgG1 and IgG2a isotype ASCs 1/15 volume of the original 4-day spleen or lymph node cultures was added to the slide. After 12 hours of incubation, slides were washed with PBS and then with PBS-Tween. Labeling antibodies (anti-mouse IgG1-A488 (Invitrogen), anti-mouse IgG2a-Cy5 (SouthernBiotech), anti-mouse IgM-A647 (Invitrogen), and anti-mouse IgG-A488 (Invitrogen)) were diluted 5000-fold in PBS containing 5% BSA (Sigma-Aldrich) and 0.05% Tween 20 (BSA-PBS-Tween). After incubation with the labeling antibodies for 1 h at RT, slides were washed with PBS-Tween, arrays were dried and scanned with an Axon GenePix 4300A scanner, and data were analyzed with ImageJ 1.43 M software or with visual inspection.

### 2.7. Statistical Analysis

Statistical difference was calculated using two-tailed Wilcoxon signed rank test in case of flow cytometric data and two-tailed permutation test was applied for fluorescent ELISPOT results. The permutation test was performed as follows: Values from the two groups to be compared were randomly reassigned to two groups and the difference between the group means was calculated. Distribution of 5000 randomizations was drawn and the two-tailed *P* value corresponding to the real sample assignments was determined. The arithmetic mean of 50 such *P* values was accepted as the probability of alpha error. For simplicity, only the results of the comparison of OVA versus OVA + ODN treatments are indicated.

## 3. Results

### 3.1. Non-CpG ODNs Enhance Early Activation Events upon Antigen Presentation

**Figure 1:**
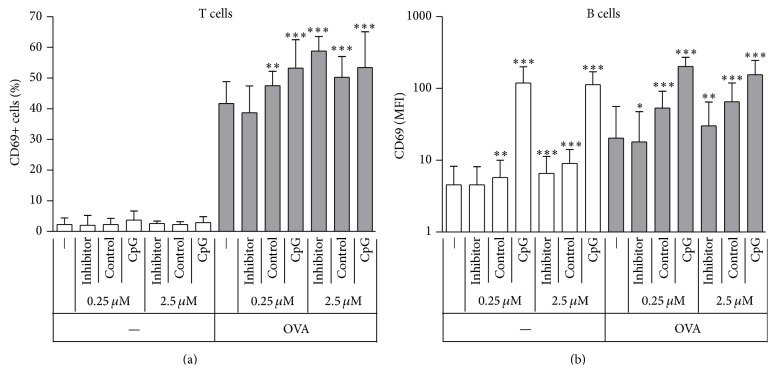
Non-CpG ODN enhances CD69 expression in T and B cells engaging in cognate interaction. DO11.10 splenocytes were incubated with different combinations of ODNs and OVA; after 24 hours CD69 expression was measured on T and B cell populations by flow cytometry ((a)-(b)). Medians and interquartile ranges of 13 independent experiments are shown. Asterisks indicate significant difference from the group receiving no ODN at all, within the respective OVA treatment group. MFI: mean fluorescence intensity; ^*∗*^ *P* < 0.05; ^*∗∗*^ *P* < 0.01; ^*∗∗∗*^ *P* < 0.001.

In order to extend earlier observations showing costimulation of T cells by non-CpG ODN [13], we utilized an antigen presentation system based on the transgenic expression of ovalbumin specific T cell receptor. Helper CD4 positive T cells from the DO11.10 mouse strain recognize an ovalbumin peptide sequence (referred to as OVA from here on). This peptide, displayed on MHCII of APCs served as TCR stimulus in our experiments. We used suspensions of splenocytes, which due to their abundance B cells (around 50% of all splenic white blood cells) strongly contribute as APC, especially when peptides that are taken up by pinocytosis and require no further processing are used as antigen. The concentration of OVA was set to 25 nM, which confers suboptimal T cell activation (Figure 1(a)) and thus enabled us to observe modulation of the outcome of antigen presentation. Along the same lines of thought, modulating ODNs were used at 0.25 *µ*M and 2.5 *µ*M concentrations, at which non-CpG ODNs (Inhibitor and Control) were found to only modestly activate B cells, in contrast to CpG ODN (Figure 1(b)).

After 24 hours of incubation about 40% of T cells expressed the early activation marker CD69 in the presence of OVA (Figure 1(a)). This basic activation was enhanced not only by coincubation with CpG ODN but also by Control and at higher concentration, Inhibitor ODN, resulting in 50–60% of the T cells being activated (Figure 1(a)). In a similar fashion, the presence of OVA resulted in increased CD69 expression on B cells, due to cognate interactions with T cells during antigen presentation (Figure 1(b)). While CpG strongly activated B cells without OVA, inducing more than tenfold increase in CD69 expression, this effect was not further intensified by the addition of antigen. In contrast, non-CpG Control ODN showed improved enhancing effects in the presence of OVA, implying the need for T cell help for the effect to take place. Thus, non-CpG ODNs enhance the activation of both T and B lymphocytes when these cells engage in cognate interaction.

### 3.2. Non-CpG ODN Effects on Late Activation Events

After receiving appropriate activation stimuli lymphocytes express various surface markers and start to differentiate and divide. To better understand the enhancing effects of non-CpG ODN exerted on T and B cells upon cognate interaction, we looked at two late activation events: expression of MHC class II on B cells and the synthesis of novel DNA as a preparation for cell division in both B and T cells.

**Figure 2:**
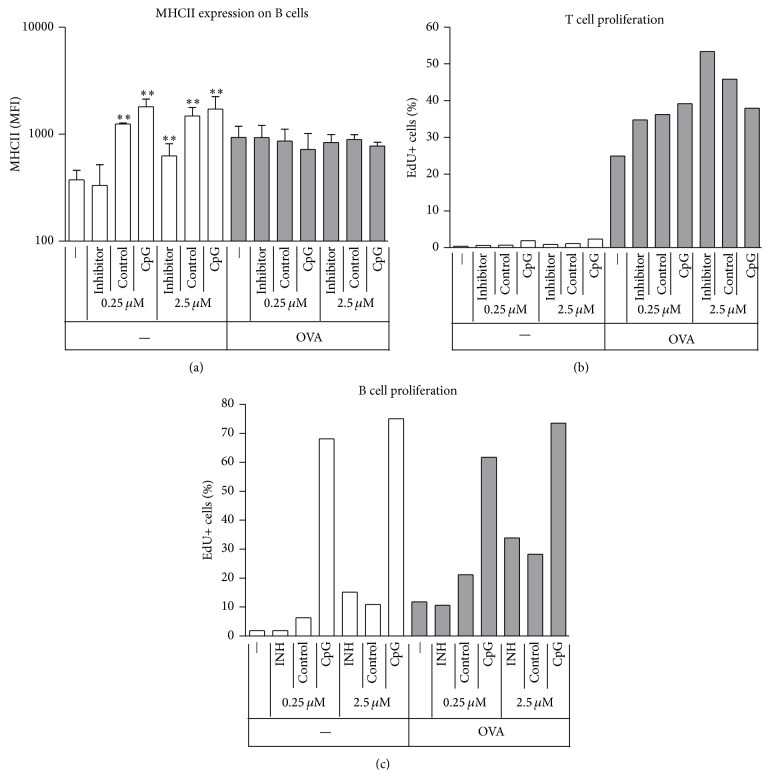
Effects of non-CpG ODN on late activation events. Spleen cells were incubated with the different combinations of ODNs and OVA as indicated. For MHCII expression measurements cells were harvested and stained for flow cytometry after 4 days (a). Medians and interquartile ranges of 9 independent experiments are shown. Asterisks indicate significant difference from the group receiving no ODN at all, within the respective OVA treatment group. MFI: mean fluorescence intensity; ^*∗*^ *P* < 0.05; ^*∗∗*^ *P* < 0.01; ^*∗∗∗*^ *P* < 0.001. For proliferation studies EdU was added on day 1 and left for incorporation for another day. The percentage of divided cells was then measured by flow cytometric analysis of EdU incorporation ((b), (c)). Results shown are from a single experiment; identical results were observed when EdU was added after 2 days.

Interestingly, the expression of MHCII did not reflect our observations with CD69. Non-CpG ODN triggered the increase of MHCII in the absence of OVA (both low and high concentrations of Control and high concentrations of Inhibitor) but had no effects in the presence of OVA (Figure 2(a)). As expected, proliferation of T cells, expressed as the percentage of cells that incorporated the nucleotide analogue EdU, was only observed in the presence of OVA (Figure 2(b)). Proliferation of both T and B cells reflected the pattern of early activation marker expression (Figure 2(c)). Thus, non-CpG ODN alone induced moderate increase of B cell MHCII expression and synergized with OVA to increase the number of dividing B cells.

### 3.3. Modulation of Differentiation into Antibody Secreting Cells (ASC) by Non-CpG ODN

B cells differentiate into antibody secreting plasmablasts and plasma cells when they receive proper stimuli. This stimulus may include T cell help, involving MHCII-TCR interactions along with the engagement of coreceptors but may also take place without T cell help. We examined the effects of non-CpG ODN on B cell differentiation into ASCs by measuring the number of cells producing antibodies of various isotypes. Using the very same experimental setup of coincubation of splenocytes with or without OVA and with the addition of ODN, after 4 days of culture the cells were transferred into wells coated with antibodies capturing light chains.

**Figure 3:**
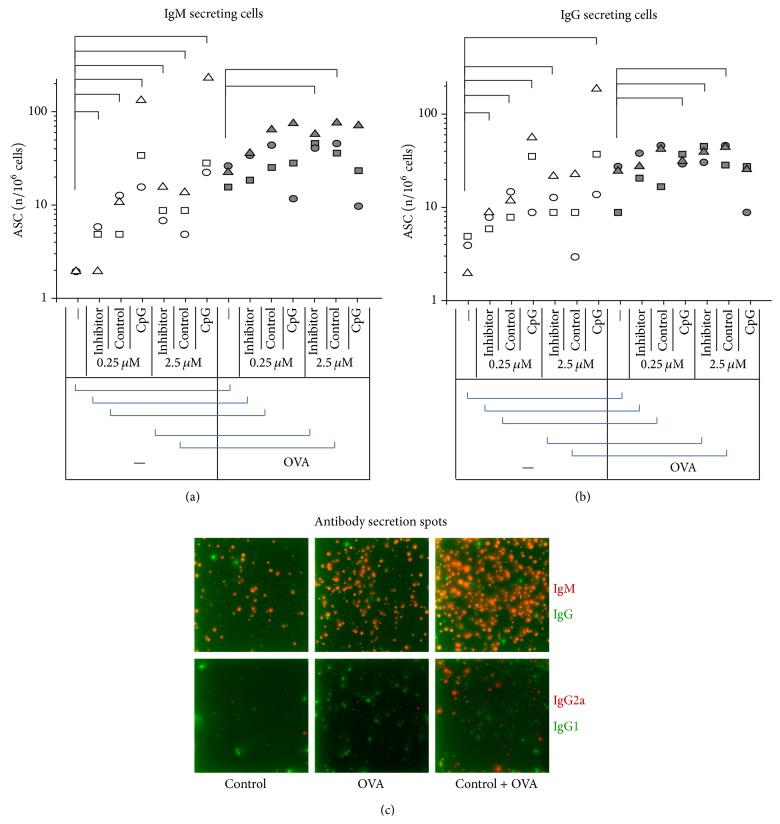
Enhancement of ASC formation by non-CpG ODN. Cells were incubated with the different combinations of ODNs and OVA as indicated; then after 4 days the cultures were transferred onto nitrocellulose-covered slides coated with light chain capture antibodies. After 10 hours of incubation, slides were washed and IgM (a) and IgG (b) spots produced by ASCs were detected using labeled antibodies and a fluorescent scanner. A representative fluorescent spot experiment is shown in (c). Statistical significance was calculated using two-tailed permutation test. Treatment pairs with significant differences (*P* < 0.05) are indicated by connecting brackets; thin lines for within OVA treatment group comparisons and thick lines for OVA treatment effects. ASC: antibody secreting cell.

The number of both IgM and IgG-producing ASCs was higher in the presence of Inhibitor or Control ODNs, as well as in the presence of CpG ODN (Figures 3(a) and 3(b)). This was true in both absence and presence of OVA; that is when T cell help was provided for B cells, implying that not only CpG but also non-CpG ODN promoted differentiation of B cells into ASC. Importantly, pairwise comparisons of non-CpG but not CpG treatments with and without OVA showed additive effects (Figures 3(a) and 3(b)).

The same trends were observed in the case of IgG1- and IgG2a-producing cells, but differences were not statistically significant (a representative measurement is shown in Figure 3(c)). Non-CpG ODNs thus enhance antibody production and isotype switching during cognate B cell-T cell interactions.

### 3.4. Cognate Interaction Is Required for Non-CpG Modulation of T Cell Induced Antibody Production

**Figure 4:**
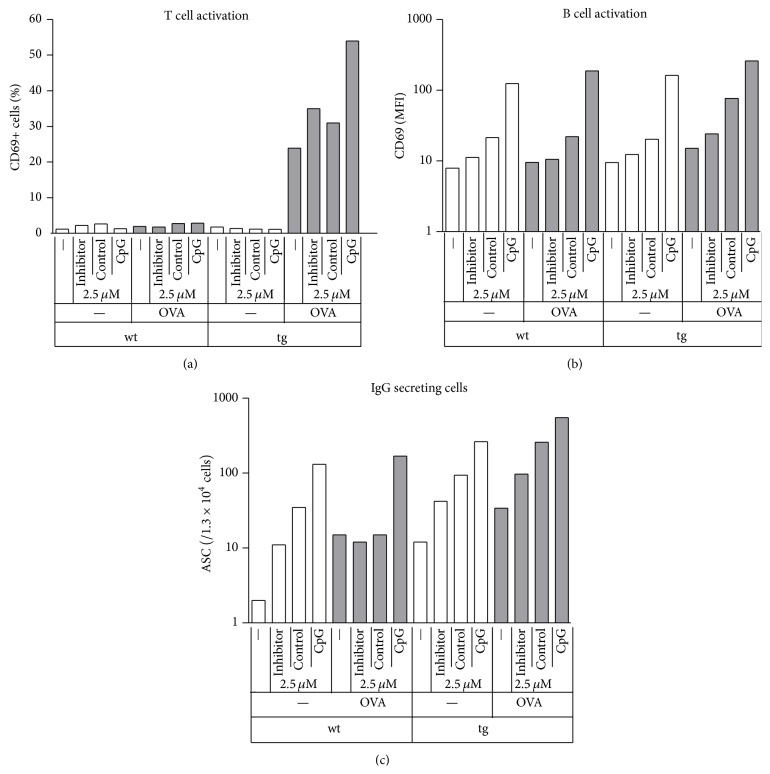
No synergism between OVA and ODN treatments when cognate T cells are not present. Wild type (wt) or TCR transgenic (tg) splenic cells were incubated with different combinations of ODN and OVA for 1 day ((a), (b)) or 4 days (c). After 1 day CD69 expression was measured on B and T cell populations by flow cytometry ((a), (b)). Cells from the 4-day cultures were washed and then transferred onto nitrocellulose-covered slides that were previously coated with light chain capture antibodies. After 10 h of incubation, slides were washed and spots indicating IgG producing ASCs were detected using fluorescently labeled antibodies and a fluorescent scanner (c). Results represent a single experiment.

To further confirm that non-CpG ODN synergize with T cell derived stimuli in enhancing antibody production and isotype switching in B cells, we repeated experiments using wild type (wt) mice. While some OVA specific T cells may be present in naïve wt mice, their numbers are expected to be so low as to be negligible (1 : 10 000 or lower). Indeed, the percentage of CD69 positive T cells was unaffected by the addition of OVA in wt mice (Figure 4(a)). In the absence of OVA, the pattern of CD69 expression in B cells was similar in wt and DO11.10 mice (Figure 4(b)). The higher concentration of non-CpG ODN used here induced modest elevation of CD69 in both strains. The presence of OVA induced further increase only in DO11.10 mice. In a similar manner, the presence of OVA enhanced IgG production only in transgenic mice (Figure 4(c)). Therefore, it is not OVA itself but the interaction of B cells presenting the peptide to T cells in its presence that further increases mutual activation of the cells and enhances antibody production and isotype switching.

## 4. Discussion

Efficient vaccination presents two challenges to the immunologist: one is finding the appropriate antigen that will stimulate the ideal B and T lymphocyte clones for achieving pathogen recognition, neutralization, and removal; the other is the formulation and administration of the antigen, in a way that the optimal costimulation is provided for those specific lymphocyte clones. Adjuvants promote these latter events, where a delicate balance should be reached between overstimulation, causing unacceptable side effects, and understimulation, leading to poor vaccination response. Immunostimulatory oligodeoxynucleotides are potent adjuvants [14], acting at least partly via the TLR9 pathway on a number of different cell types [15–17]. Depending on the dosage and administration route, unwanted systemic effects may be triggered, which call for caution regarding their human usage.

The exact mechanism by which different ODNs exert their costimulatory effect on T cells is not fully elucidated yet, as there is incongruity between reports regarding the dependence of ODN effects on the presence of TLR9 and MyD88 [13, 18]. Therefore, it is not clear if there are other receptors and mechanisms that could be responsible for the costimulatory potential of non-CpG ODN.

Our approach of examining non-CpG ODN with moderate adjuvant effects [19, 20] was an extension of observations on the T cell stimulatory properties of such ODN [13]. Landrigan et al. used an artificial T cell stimulation system with CD3 and CD28 triggered activation. In contrast, we set up an experimental system where APCs (predominantly B cells) in the spleen and lymph node suspensions present to transgenic T cells their cognate antigen (OVA). Activated T cells then provide help for B cells for activation and maturation. This approach has the advantages of being closer to T cell activation upon antigen presentation* in vivo* and of being suitable for studying the effects on B cells, as well. The disadvantage is that ODN effects exerted on B or T cells cannot be distinguished.

As expected based on previous observations, T cells were not activated when treated by different ODN alone, even when B cells were activated by CpG ODN treatment (Figure 1). When antigen and ODN treatments were combined, synergism was observed in every aspect studied, ranging from early activation events (Figure 1) through late activation events (Figure 2) to promotion of B cell differentiation into ASCs (Figure 3). This effect was not due to separated APC- and T cell activating effects of ODNs, as non-CpG ODNs further increased APC activation only when given in combination with T cell antigen. The synergism was not observed when wt cells were used, ruling out the possibility that the OVA peptide itself or contamination in the peptide preparation would be responsible for the enhancement (Figure 4).

ODNs that do not or only moderately activate B cells could be used in combination with antigen to achieve B cell activation, differentiation and isotype switching via cognate interaction with antigen specific T cells. From our experiments we cannot categorically conclude whether non-CpG ODN showed antigen presentation enhancing effects via B cells, T cells, or both. The non-CpG ODN dosage we utilized showed modest effects on early B cell activation in itself and enhanced MHCII expression and ASC formation. The contribution of this effect on B cells, therefore, should be taken into account. The experiments of Landrigan et al. clearly showed a costimulatory effect of non-CpG ODN on T cells [13]. Our proposition is that non-CpG ODNs act on both B and T cells, and this effect becomes prominent when these two cell types engage in cognate interaction during antigen presentation. As we used a small peptide antigen, we assume that antigen was taken up potentially by all B cells and BCR-mediated uptake was not needed. Therefore, presumably all B cell clones presented antigen to T cells and were all partners of helper functions of costimulated tg T cells. It remains to be examined whether antigen uptake via the BCR would restrict the B cell clones affected by non-CpG treatment in our experimental system to those that specifically recognize antigen.

Class switching in murine B cells follows a pattern that is the function of the number of cell divisions [21]. Our experiments tracking dividing B cells (Figure 2(c)) showed nice correlation with the number of IgG producing cells (Figures 3(b) and 3(c)), suggesting that non-CpG ODN synergism with T cell help enhanced isotype switching via the augmentation of proliferation.

Although there is evidence concerning the requirement of CpG DNA-mediated PI-3 kinase activation via T cell-expressed MyD88 in a CD4^+^ T cell-dependent response* in vivo* [18], the mechanism of action and possible role of other types of ODN is not elucidated. Our study focused on the B cell response, following T cell costimulation with different CpG and non-CpG ODN, and verifies their impact on T cell mediated B cell responses* in vitro*. Further studies are needed to assess the effect of ODN-costimulation of T cells* in vivo*, preferentially using ODNs that do not stimulate APC. Studying human samples would also be important, as murine and human TLR expression profile and certain TLR signaling pathways show species-specific differences.

## 5. Concluding Remarks

Taking into account that CpG ODN induce cell activation irrespective of the coadministration of antigen, we propose that nonstimulatory ODNs are promising candidates as possible vaccine adjuvants, because they costimulate only T cells responding to the administered antigen and in turn provide help for appropriate B cell clones presenting the antigen, a feature that could provide a more restricted and focused B cell activation and differentiation profile.

## Abbreviations

APC: Antigen-presenting cell
ASC: Antibody-secreting cell
CpG: Cytosine guanine dinucleotide-containing unmethylated DNA motif (in this study: ODN 1668)
Control: Control oligonucleotide (in this study: ODN 1720)
IFA: Incomplete Freund's adjuvant
Inhibitor: TLR9 antagonist oligonucleotide (in this study: ODN 2088)
LN: Lymph node
MFI: Mean fluorescence intensity
ODN: Oligodeoxynucleotide
OVA: Ovalbumin peptide (in this study: Biotin-KISQAVHAAHAEINEAGR)
PS: Phosphorothioate.
